# 4-(8-Hydr­oxy-3-methyl-1,4-dioxo-1,4-dihydro-2-naphth­yl)butanoic acid

**DOI:** 10.1107/S1600536809040021

**Published:** 2009-10-17

**Authors:** Yan-Fei Wang, Huang Tang, Yan-Cheng Liu, Zhen-Feng Chen, Hong Liang

**Affiliations:** a School of Chemistry and Chemical Engineering, Central South University, Changsha 410083, People’s Republic of China; b Key Laboratory for the Chemistry and Molecular Engineering of Medicinal Resources (Ministry of Education), Guangxi Normal University, Guilin 541004, People’s Republic of China

## Abstract

In the title compound, C_15_H_14_O_5_, an intramolecular O—H⋯O hydrogen bond occurs.  In the crystal, the molecules form inversion dimers linked by pairs of O—H⋯O bonds, which are further linked by C—H⋯O interactions.

## Related literature

For the synthesis and biological properties of the title compound, see: Salmon-Chemin *et al.* (2001 ▶). For crystal structures of similar compounds, see: Vijayalakshmi *et al.* (1987 ▶); Ghouse & Rao (1974 ▶).
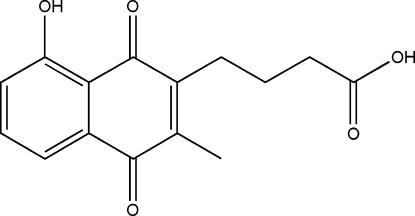


## Experimental

### 

#### Crystal data

C_15_H_14_O_5_
*M* *_r_* = 274.26
Monoclinic, 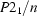

*a* = 10.881 (3) Å
*b* = 9.973 (2) Å
*c* = 12.705 (3) Å
β = 106.936 (5)°
*V* = 1319.0 (6) Å^3^
*Z* = 4
Mo *K*α radiation
μ = 0.10 mm^−1^
*T* = 293 K
0.45 × 0.30 × 0.24 mm

#### Data collection

Rigaku Mercury CCD diffractometer
Absorption correction: multi-scan (REQAB: Jacobson, 1998 ▶) *T*_min_ = 0.734, *T*_max_ = 0.975
11416 measured reflections
2405 independent reflections
1779 reflections with *I* > 2σ(*I*)
*R*_int_ = 0.038

#### Refinement

*R*[*F* ^2^ > 2σ(*F* ^2^)] = 0.073
*wR*(*F* ^2^) = 0.198
*S* = 1.09
2405 reflections
184 parameters
H-atom parameters constrained
Δρ_max_ = 0.19 e Å^−3^
Δρ_min_ = −0.25 e Å^−3^

### 

Data collection: *CrystalClear* (Rigaku, 1999 ▶); cell refinement: *CrystalClear*; data reduction: *CrystalStructure* (Rigaku/MSC & Rigaku, 2000 ▶); program(s) used to solve structure: *SHELXS97* (Sheldrick, 2008 ▶); program(s) used to refine structure: *SHELXL97* (Sheldrick, 2008 ▶); molecular graphics: *SHELXTL* (Sheldrick, 2008 ▶); software used to prepare material for publication: *SHELXTL*.

## Supplementary Material

Crystal structure: contains datablocks I, global. DOI: 10.1107/S1600536809040021/pk2192sup1.cif

Structure factors: contains datablocks I. DOI: 10.1107/S1600536809040021/pk2192Isup2.hkl

Additional supplementary materials:  crystallographic information; 3D view; checkCIF report

## Figures and Tables

**Table 1:** Hydrogen-bond geometry (Å, °)

*D*—H⋯*A*	*D*—H	H⋯*A*	*D*⋯*A*	*D*—H⋯*A*
C3—H3⋯O2^i^	0.93	2.43	3.315 (4)	160
O5—H5⋯O4^ii^	0.82	1.77	2.589 (3)	174
O1—H1⋯O3	0.82	1.87	2.582 (3)	145

Symmetry codes: (i) 
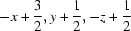
; (ii) 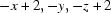.

**Fig. 1.**
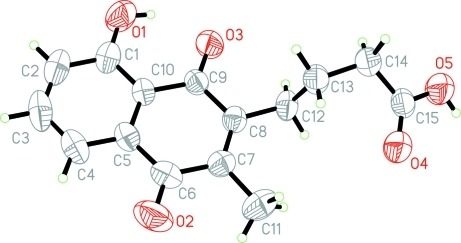
The molecular structure showing the atom-labelling scheme. Displacement ellipsoids are drawn at the 50% probability level.

**Fig. 2.**
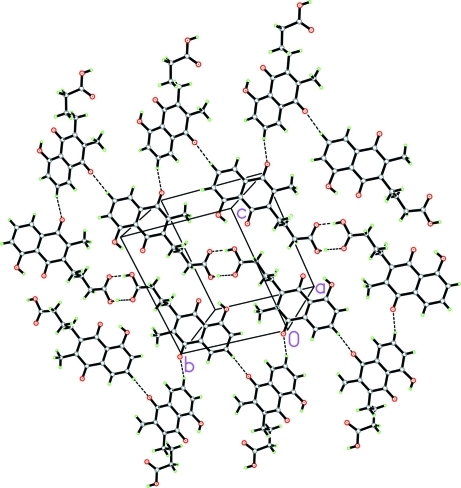
Packing of molecules roughly down the [100] direction showing the two-dimensional network of molecules. Hydrogen bonds are shown as dashed lines.

## supplementary crystallographic information

### Comment

Plumbagin is a potent toxic natural product extracted from Plumbago Zeylanica *L*. (Plumbaginaceae), which has been used in China as well as other Asian countries for the treatment of rheumatoid arthritis, dysmenorrhea, injury by bumping, and even cancer. The title compound is a 2-substituted 1,4-naphthoquinone derivative. Its synthesis has been reported by Salmon-Chemin *et al.*(2001), we now report its structure. The molecular structure of the title compound is shown in Fig.1. The bond lengths and angles of the napthoquinone molecule are normal and comparable to those of plumbagin (Ghouse & Rao, 1974; Vijayalakshmi, *et al.*, 1987). Geometric parameters for the butanoic acid group are also normal. As shown in Fig.2, a two-dimensional network is generated *via* intermolecular hydrogen bond interactions involving C—H···O, O—H···O.

### Experimental

0.2 mmol compound were dissolved in 10 ml methanol and 10 ml CH_2_Cl_2_. The resulting red solution was filtered. The filtrate was allowed to sit under ambient conditions for two weeks, dark-red block crystals were obtained.

### Refinement

The H bound to C atoms of naphthoquinone, and to C(11) as well as to C(12)—C(14) were treated as riding, with C—H distances of 0.93, 0.96 and 0.97Å with U_iso_(H) = 1.2U_eq_(C), respectively. Hydroxyl O—H distances were set to 0.82Å and were refined as riding with U_iso_(H) = 1.5U_eq_(O).

### Crystal data

**Table:**

C_15_H_14_O_5_	*F*(000) = 576
*M**_r_* = 274.26	*D*_x_ = 1.381 Mg m^−^^3^
Monoclinic, *P*2_1_/*n*	Mo *K*α radiation, λ = 0.71070 Å
Hall symbol: -P 2yn	Cell parameters from 3725 reflections
*a* = 10.881 (3) Å	θ = 3.4–25.3°
*b* = 9.973 (2) Å	µ = 0.10 mm^−^^1^
*c* = 12.705 (3) Å	*T* = 293 K
β = 106.936 (5)°	Block, dark-red
*V* = 1319.0 (6) Å^3^	0.45 × 0.30 × 0.24 mm
*Z* = 4	

### Data collection

**Table:**

Rigaku Mercury CCD diffractometer	2405 independent reflections
Radiation source: fine-focus sealed tube	1779 reflections with *I* > 2σ(*I*)
graphite	*R*_int_ = 0.038
Detector resolution: 7.31 pixels mm^-1^	θ_max_ = 25.4°, θ_min_ = 3.4°
ω scans	*h* = −13→13
Absorption correction: multi-scan (REQAB: Jacobson, 1998)	*k* = −11→12
*T*_min_ = 0.734, *T*_max_ = 0.975	*l* = −13→15
11416 measured reflections	

### Refinement

**Table:**

Refinement on *F*^2^	Primary atom site location: structure-invariant direct methods
Least-squares matrix: full	Secondary atom site location: difference Fourier map
*R*[*F*^2^ > 2σ(*F*^2^)] = 0.073	Hydrogen site location: inferred from neighbouring sites
*wR*(*F*^2^) = 0.198	H-atom parameters constrained
*S* = 1.09	*w* = 1/[σ^2^(*F*_o_^2^) + (0.0888*P*)^2^ + 0.5298*P*] where *P* = (*F*_o_^2^ + 2*F*_c_^2^)/3
2405 reflections	(Δ/σ)_max_ < 0.001
184 parameters	Δρ_max_ = 0.19 e Å^−^^3^
0 restraints	Δρ_min_ = −0.25 e Å^−^^3^

### Special details

**Table:**

Geometry. All e.s.d.'s (except the e.s.d. in the dihedral angle between two l.s. planes) are estimated using the full covariance matrix. The cell e.s.d.'s are taken into account individually in the estimation of e.s.d.'s in distances, angles and torsion angles; correlations between e.s.d.'s in cell parameters are only used when they are defined by crystal symmetry. An approximate (isotropic) treatment of cell e.s.d.'s is used for estimating e.s.d.'s involving l.s. planes.
Refinement. Refinement of *F*^2^ against ALL reflections. The weighted *R*-factor *wR* and goodness of fit *S* are based on *F*^2^, conventional *R*-factors *R* are based on *F*, with *F* set to zero for negative *F*^2^. The threshold expression of *F*^2^ > σ(*F*^2^) is used only for calculating *R*-factors(gt) *etc*. and is not relevant to the choice of reflections for refinement. *R*-factors based on *F*^2^ are statistically about twice as large as those based on *F*, and *R*- factors based on ALL data will be even larger.

### Fractional atomic coordinates and isotropic or equivalent isotropic displacement parameters (Å^2^)

**Table:**

	*x*	*y*	*z*	*U*_iso_*/*U*_eq_	
O1	0.9902 (2)	0.8451 (2)	0.6287 (2)	0.0868 (8)	
H1	1.0115	0.8001	0.6851	0.130*	
O2	0.7285 (3)	0.3526 (3)	0.4035 (2)	0.0943 (9)	
O3	1.0036 (2)	0.6313 (2)	0.74605 (17)	0.0752 (7)	
O4	0.9090 (2)	0.1025 (2)	0.90860 (19)	0.0749 (7)	
O5	1.0253 (3)	0.1478 (2)	1.08016 (19)	0.0808 (8)	
H5	1.0411	0.0674	1.0806	0.121*	
C1	0.9241 (3)	0.7683 (3)	0.5446 (3)	0.0619 (8)	
C2	0.8795 (3)	0.8269 (4)	0.4409 (3)	0.0741 (10)	
H2	0.8964	0.9169	0.4317	0.089*	
C3	0.8115 (3)	0.7538 (4)	0.3534 (3)	0.0782 (11)	
H3	0.7836	0.7940	0.2846	0.094*	
C4	0.7827 (3)	0.6200 (4)	0.3646 (2)	0.0665 (9)	
H4	0.7346	0.5716	0.3040	0.080*	
C5	0.8260 (2)	0.5593 (3)	0.4661 (2)	0.0516 (7)	
C6	0.7964 (3)	0.4165 (3)	0.4808 (2)	0.0587 (8)	
C7	0.8472 (3)	0.3520 (3)	0.5893 (2)	0.0523 (7)	
C8	0.9164 (2)	0.4235 (3)	0.6766 (2)	0.0475 (7)	
C9	0.9428 (3)	0.5675 (3)	0.6644 (2)	0.0490 (7)	
C10	0.8974 (2)	0.6322 (3)	0.5572 (2)	0.0480 (7)	
C11	0.8153 (3)	0.2063 (3)	0.5962 (3)	0.0781 (10)	
H11A	0.8895	0.1597	0.6407	0.117*	
H11B	0.7897	0.1682	0.5237	0.117*	
H11C	0.7462	0.1978	0.6287	0.117*	
C12	0.9674 (3)	0.3671 (3)	0.7905 (2)	0.0565 (8)	
H12A	1.0433	0.4168	0.8303	0.068*	
H12B	0.9923	0.2744	0.7859	0.068*	
C13	0.8682 (3)	0.3741 (3)	0.8535 (2)	0.0593 (8)	
H13A	0.8385	0.4658	0.8531	0.071*	
H13B	0.7949	0.3189	0.8165	0.071*	
C14	0.9219 (3)	0.3269 (3)	0.9717 (3)	0.0640 (8)	
H14A	0.9995	0.3771	1.0063	0.077*	
H14B	0.8600	0.3470	1.0111	0.077*	
C15	0.9521 (3)	0.1824 (3)	0.9828 (3)	0.0582 (8)	

### Atomic displacement parameters (Å^2^)

**Table:**

	*U*^11^	*U*^22^	*U*^33^	*U*^12^	*U*^13^	*U*^23^
O1	0.1058 (19)	0.0542 (14)	0.0918 (18)	−0.0175 (13)	0.0155 (15)	0.0019 (12)
O2	0.1062 (19)	0.106 (2)	0.0626 (15)	−0.0342 (16)	0.0118 (14)	−0.0271 (14)
O3	0.0995 (17)	0.0634 (13)	0.0507 (12)	−0.0164 (12)	0.0031 (12)	−0.0043 (10)
O4	0.0928 (17)	0.0626 (14)	0.0654 (15)	0.0010 (12)	0.0167 (13)	0.0090 (11)
O5	0.1021 (18)	0.0667 (15)	0.0655 (15)	0.0022 (14)	0.0119 (13)	0.0084 (11)
C1	0.0604 (17)	0.0583 (19)	0.067 (2)	0.0009 (15)	0.0189 (16)	0.0156 (16)
C2	0.074 (2)	0.065 (2)	0.087 (3)	0.0133 (17)	0.030 (2)	0.0290 (19)
C3	0.069 (2)	0.106 (3)	0.064 (2)	0.024 (2)	0.0276 (18)	0.040 (2)
C4	0.0576 (18)	0.097 (3)	0.0443 (16)	0.0080 (17)	0.0132 (14)	0.0075 (16)
C5	0.0471 (15)	0.0657 (19)	0.0428 (15)	0.0011 (13)	0.0143 (13)	0.0042 (13)
C6	0.0525 (16)	0.073 (2)	0.0518 (17)	−0.0070 (15)	0.0166 (14)	−0.0131 (15)
C7	0.0511 (15)	0.0492 (16)	0.0593 (17)	−0.0005 (13)	0.0204 (14)	−0.0020 (13)
C8	0.0454 (14)	0.0504 (16)	0.0496 (15)	0.0017 (12)	0.0183 (12)	0.0047 (12)
C9	0.0519 (15)	0.0507 (16)	0.0427 (15)	−0.0022 (13)	0.0110 (13)	0.0008 (12)
C10	0.0479 (15)	0.0533 (16)	0.0429 (15)	0.0017 (12)	0.0134 (12)	0.0071 (12)
C11	0.079 (2)	0.0543 (19)	0.104 (3)	−0.0133 (17)	0.031 (2)	−0.0053 (18)
C12	0.0590 (17)	0.0578 (18)	0.0545 (17)	0.0092 (14)	0.0195 (14)	0.0184 (13)
C13	0.0690 (19)	0.0570 (18)	0.0561 (17)	0.0093 (14)	0.0247 (15)	0.0090 (14)
C14	0.080 (2)	0.060 (2)	0.0567 (18)	0.0069 (16)	0.0276 (16)	0.0067 (14)
C15	0.0665 (18)	0.062 (2)	0.0472 (16)	−0.0049 (15)	0.0177 (14)	0.0079 (14)

### Geometric parameters (Å, °)

**Table:**

O1—C1	1.341 (4)	C7—C8	1.349 (4)
O1—H1	0.8200	C7—C11	1.502 (4)
O2—C6	1.222 (3)	C8—C9	1.482 (4)
O3—C9	1.232 (3)	C8—C12	1.500 (4)
O4—C15	1.220 (4)	C9—C10	1.457 (4)
O5—C15	1.307 (4)	C11—H11A	0.9600
O5—H5	0.8200	C11—H11B	0.9600
C1—C2	1.393 (4)	C11—H11C	0.9600
C1—C10	1.407 (4)	C12—C13	1.521 (4)
C2—C3	1.355 (5)	C12—H12A	0.9700
C2—H2	0.9300	C12—H12B	0.9700
C3—C4	1.387 (5)	C13—C14	1.520 (4)
C3—H3	0.9300	C13—H13A	0.9700
C4—C5	1.377 (4)	C13—H13B	0.9700
C4—H4	0.9300	C14—C15	1.476 (4)
C5—C10	1.395 (4)	C14—H14A	0.9700
C5—C6	1.484 (4)	C14—H14B	0.9700
C6—C7	1.475 (4)		
			
C1—O1—H1	109.5	C5—C10—C9	120.0 (3)
C15—O5—H5	109.5	C1—C10—C9	120.5 (3)
O1—C1—C2	118.1 (3)	C7—C11—H11A	109.5
O1—C1—C10	122.8 (3)	C7—C11—H11B	109.5
C2—C1—C10	119.1 (3)	H11A—C11—H11B	109.5
C3—C2—C1	120.4 (3)	C7—C11—H11C	109.5
C3—C2—H2	119.8	H11A—C11—H11C	109.5
C1—C2—H2	119.8	H11B—C11—H11C	109.5
C2—C3—C4	121.3 (3)	C8—C12—C13	111.8 (2)
C2—C3—H3	119.3	C8—C12—H12A	109.3
C4—C3—H3	119.3	C13—C12—H12A	109.3
C5—C4—C3	119.5 (3)	C8—C12—H12B	109.3
C5—C4—H4	120.3	C13—C12—H12B	109.3
C3—C4—H4	120.3	H12A—C12—H12B	107.9
C4—C5—C10	120.2 (3)	C14—C13—C12	112.2 (2)
C4—C5—C6	120.8 (3)	C14—C13—H13A	109.2
C10—C5—C6	119.0 (2)	C12—C13—H13A	109.2
O2—C6—C7	119.9 (3)	C14—C13—H13B	109.2
O2—C6—C5	120.1 (3)	C12—C13—H13B	109.2
C7—C6—C5	120.0 (2)	H13A—C13—H13B	107.9
C8—C7—C6	120.4 (3)	C15—C14—C13	114.1 (3)
C8—C7—C11	123.1 (3)	C15—C14—H14A	108.7
C6—C7—C11	116.5 (3)	C13—C14—H14A	108.7
C7—C8—C9	120.3 (2)	C15—C14—H14B	108.7
C7—C8—C12	123.8 (3)	C13—C14—H14B	108.7
C9—C8—C12	115.9 (2)	H14A—C14—H14B	107.6
O3—C9—C10	120.8 (3)	O4—C15—O5	123.4 (3)
O3—C9—C8	119.0 (2)	O4—C15—C14	122.6 (3)
C10—C9—C8	120.2 (2)	O5—C15—C14	113.9 (3)
C5—C10—C1	119.4 (3)		
			
O1—C1—C2—C3	−179.5 (3)	C7—C8—C9—C10	−1.9 (4)
C10—C1—C2—C3	−0.1 (5)	C12—C8—C9—C10	−180.0 (2)
C1—C2—C3—C4	1.0 (5)	C4—C5—C10—C1	0.3 (4)
C2—C3—C4—C5	−1.2 (5)	C6—C5—C10—C1	−178.9 (3)
C3—C4—C5—C10	0.5 (4)	C4—C5—C10—C9	179.9 (3)
C3—C4—C5—C6	179.7 (3)	C6—C5—C10—C9	0.7 (4)
C4—C5—C6—O2	−3.0 (4)	O1—C1—C10—C5	178.8 (3)
C10—C5—C6—O2	176.2 (3)	C2—C1—C10—C5	−0.5 (4)
C4—C5—C6—C7	178.0 (3)	O1—C1—C10—C9	−0.7 (4)
C10—C5—C6—C7	−2.8 (4)	C2—C1—C10—C9	179.9 (3)
O2—C6—C7—C8	−176.4 (3)	O3—C9—C10—C5	−178.5 (3)
C5—C6—C7—C8	2.6 (4)	C8—C9—C10—C5	1.7 (4)
O2—C6—C7—C11	2.5 (4)	O3—C9—C10—C1	1.0 (4)
C5—C6—C7—C11	−178.5 (3)	C8—C9—C10—C1	−178.8 (3)
C6—C7—C8—C9	−0.2 (4)	C7—C8—C12—C13	−85.5 (3)
C11—C7—C8—C9	−179.1 (3)	C9—C8—C12—C13	92.5 (3)
C6—C7—C8—C12	177.7 (2)	C8—C12—C13—C14	−175.7 (2)
C11—C7—C8—C12	−1.1 (4)	C12—C13—C14—C15	−67.6 (4)
C7—C8—C9—O3	178.3 (3)	C13—C14—C15—O4	−17.6 (5)
C12—C8—C9—O3	0.2 (4)	C13—C14—C15—O5	164.6 (3)

### Hydrogen-bond geometry (Å, °)

**Table:**

*D*—H···*A*	*D*—H	H···*A*	*D*···*A*	*D*—H···*A*
C3—H3···O2^i^	0.93	2.43	3.315 (4)	160
O5—H5···O4^ii^	0.82	1.77	2.589 (3)	174
O1—H1···O3	0.82	1.87	2.582 (3)	145

Symmetry codes: (i) −*x*+3/2, *y*+1/2, −*z*+1/2; (ii) −*x*+2, −*y*, −*z*+2.
